# Dynamics of vision: Grouping takes longer than crowding

**DOI:** 10.1167/jov.25.12.16

**Published:** 2025-10-08

**Authors:** Martina Morea, Michael H. Herzog, Gregory Francis, Mauro Manassi

**Affiliations:** 1 Laboratory of Psychophysics, Brain Mind Institute, École Polytechnique Fédérale de Lausanne (EPFL), Lausanne, Switzerland; 2 Department of Psychological Sciences, Purdue University, West Lafayette, IN, USA; 3 School of Psychology, University of Aberdeen, King's College, Aberdeen, United Kingdom

**Keywords:** visual crowding, temporal dynamics, perceptual grouping, neural network models, segmentation

## Abstract

Vision is often understood as a hierarchical, feedforward process, where visual processing proceeds from low-level features to high-level representations. Within tens of milliseconds, the fundamental features of the percept are established. Traditional models use this framework to explain visual crowding, where nearby elements impair target perception with minimal influence from stimulus duration. Here, we show that, at least for more complex displays, crowding involves highly dynamic processes. We determined vernier offset discrimination thresholds for different flanker configurations. In Experiment 1, for a 160-ms stimulus duration, crowding was lower for flanking Cubes/Rectangles compared to Lines, pointing toward underlying grouping processes. However, strong crowding occurred in all conditions at 20 ms, showing that grouping requires a minimum stimulus duration to occur. In Experiment 2, the crowded vernier (20 ms) was preceded by a 20-ms Cubes display. This brief preview led to uncrowding of the subsequently presented flanked vernier, but only for flankers that ungroup for longer durations (i.e., Cubes). This uncrowding effect occurred for time spans up to 1 s (Experiment 3) but could be interrupted by elements presented between the preview and the flanked vernier (Experiment 4). Our findings are well predicted by the LAMINART model, which employs recurrent segmentation processes unfolding over time to separate objects into distinct representation layers. Taken together, our novel preview effect highlights the importance of spatiotemporal grouping in crowding. In contrast to classic feedforward models, we propose that crowding is a dynamic process where multiple interpretations are modulated and gated by grouping mechanisms evolving over time.

## Introduction

In classic models of vision, a stimulus is processed in a feedforward fashion in the visual hierarchy until a conscious percept is elicited. In the early stages of processing, low-level features, such as edges, are extracted, which become the building blocks for textures, objects, and scenes in subsequent processing stages (Cichy, Pantazis, & Oliva, 2014; DiCarlo, Zoccolan, & Rust, 2012; Hubel & Wiesel, 1962; Ricci & Serre, 2020; Riesenhuber & Poggio, 1999). Deep convolutional networks have demonstrated that this feedforward approach is highly effective for object recognition (Cichy, Khosla, Pantazis, Torralba, & Oliva, 2016; He, Zhang, Ren, & Sun, 2016; Krizhevsky, Sutskever, & Hinton, 2012; Lecun, Bottou, Bengio, & Haffner, 1998; Simonyan & Zisserman, 2014). Psychophysical studies further support this idea, since humans can detect objects with a remarkable speed that does not need or allow recurrent processing (Fabre-Thorpe, Delorme, Marlot, & Thorpe, 2001; Thorpe, Fize, & Marlot, 1996). According to this view, feedback projections in the brain are mostly associated with attention, expectation, and other top-down processes that influence feedforward processing, such as prioritizing attended stimuli (for a review, see Gilbert & Li, 2013).

These classic feedforward models of vision have been the main theoretical framework to explain visual crowding, where the perception of a target object is impaired by the presence of nearby objects (Levi, 2008; Manassi & Whitney, 2018; Pelli, 2008; Strasburger, Rentschler, & Jüttner, 2011; Whitney & Levi, 2011). For instance, vernier offset discrimination accuracy significantly decreases when two vertical lines are placed on either side of the target (Levi, Klein, & Aitsebaomo, 1985; Malania, Herzog, & Westheimer, 2007; Westheimer & Hauske, 1975). Extending the presentation time of the target and flankers to several hundred milliseconds only slightly reduces the crowding effect, indicating that prolonged processing time has little impact on perception in classic crowding paradigms (Wallace, Chiu, Nandy, & Tjan, 2013). These characteristics align with the idea that the initial stages of visual processing in a feedforward fashion are most essential for perceiving stimuli under crowding conditions. Contrary to this view, here we show that a 20-ms *pre*view of flankers can lead to uncrowding for objects presented up to 1 s later. Importantly, this preview effect is configuration specific and, hence, different from priming.

We propose that a brief 20-ms preview initiates flanker processing across time, allowing their representations to be grouped away from the subsequent vernier target. Hence, visual processing can require substantial time and is fundamentally shaped by grouping processes (Bornet et al., 2021; Bornet, Doerig, Herzog, Francis, & Van Der Burg, 2021; Choung, Bornet, Doerig, & Herzog, 2021; Choung, Rashal, Kunchulia, & Herzog, 2023; Francis, Manassi, & Herzog, 2017; Herzog, 2022; Herzog, Sayim, Chicherov, & Manassi, 2015; Herzog & Manassi, 2015; Jastrzębowska, Chicherov, Draganski, & Herzog, 2021; Manassi, France, & Herzog, 2012; Manassi, Sayim, & Herzog, 2013; Manassi, Hermens, Francis, & Herzog, 2015; Manassi, Lonchampt, Clarke, & Herzog, 2016; Saarela, Sayim, Westheimer, & Herzog, 2009; Saarela, Westheimer, & Herzog, 2010; Sayim, Manassi, & Herzog, 2014; Sayim, Westheimer, & Herzog, 2010; Tiurina, Markov, Choung, Herzog, & Pascucci, 2022). What matters is not the presentation time per se but the time allocated for grouping processes. We show that this body of results can be well explained by a previously developed neural network model of visual perception, LAMINART (Francis et al., 2017; Kon & Francis, 2022; Manassi et al., 2015). In this model, recurrent processing segments the visual scene into distinct objects represented in separate segmentation layers. Crowding occurs when the target and flankers are processed in the same layer; uncrowding occurs when they are processed in different layers. Crucially, the flanker preview gives the model sufficient time to segment the vernier target separately, reducing crowding strength. These findings challenge classic feedforward models by demonstrating that visual perception is a dynamic process fundamentally shaped by grouping mechanisms that unfold over time.

## General methods

### Observers

Participants were paid students of the École Polytechnique Fédérale de Lausanne (EPFL). All had normal or corrected-to-normal vision, with a visual acuity of 1.0 (corresponding to 20/20) or better, as measured with the Freiburg Visual Acuity Test (Bach, 1996). Participants provided informed consent, were informed of the general purpose of the experiment, and were told that they could quit the experiment at any time. There were 14 observers in Experiment 1 (9 females, *M*_*age* _ = 21.9, *SD* = 2.3), 10 in Experiment 2 (4 females, *M*_*age* _ = 21.4, *SD* = 1.3), 10 in Experiment 3 (4 females, *M*_*age* _ = 23.3, *SD* = 2.0), and 12 in Experiment 4 (5 females, *M*_*age* _ = 23.0, *SD* = 2.8). Participants were not involved in more than one experiment. Participants showing exceptionally unstable performance or excessive ceiling/floor effects were excluded (Three from Experiment 1, three from Experiment 2, two from Experiment 3, three from Experiment 4; 11 in total). The experiments were approved by the local ethics committee.

### Apparatus and stimuli

Stimuli for Experiment 1 were presented on an HP-1332A XY-display equipped with a P11 phosphor and controlled by a custom-made 16-bit DA interface with custom-made software. Experiments 2 to 4 were carried out on an Asus VG248QE LCD monitor (1,920 × 1,080 pixels, 120 Hz). Stimulus programs were implemented in MATLAB (R2019b; The MathWorks, Natick, MA, USA) using the Psychophysics Toolbox (Brainard, 1997). The stimuli were white (100 cd/m^2^) on a black background (luminance below 0.3 cd/m^2^, as measured with a Minolta Luminance meter LS-100). The experimental room was dimly illuminated. Viewing distance was 75 cm.

**Figure 1.**
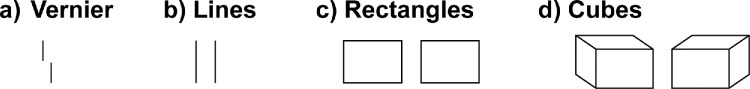
Stimuli used in the experiments. The target (**a**) was composed of two vertical lines slightly offset either to the left or to the right (vernier). The Lines consisted of two vertical lines on each side of the vernier (**b**). The Rectangles (**c**) contained the lines from the Lines condition, and the Cubes (**d**) contained the rectangles from the Rectangles condition.

We determined vernier offset discrimination thresholds for different flanker configurations. The vernier target consisted of two lines that were randomly offset to the left or right. Stimuli consisted of two vertical 40′ (arcmin) long lines separated by a vertical gap of 4′ and presented at an eccentricity of 9° (arcdeg) to the right of a red fixation dot (8′ diameter). Observers indicated the offset direction of the bottom line of the vernier. Stimuli were moved to 6° eccentricity if the participant could not perform the task (e.g., had performance at chance for every condition besides the unflanked vernier). Sixteen participants in total performed the experiment at 6° eccentricity (one for Experiment 1, four for Experiment 2, five for Experiment 3, and six for Experiment 4). Eccentricity refers to the center of the vernier stimulus. Flanker configurations were placed symmetrically to the left and right of the vernier stimulus. There were three possible flanker configurations of increasing complexity (Figure 1). Each more complex configuration is obtained by adding but not omitting elements from the simpler configurations. In the “Lines” configuration, the vernier was flanked by two vertical lines (84′, the same length as the vernier) spaced 46′ apart. In the “Rectangles” configuration, rectangles were presented to the left and right of the vernier (width = 117′, same height as the lines). In the “Cubes” configuration, perspective Cubes were presented to the left and right of the vernier (same dimensions as Rectangles, angle of oblique lines = 45°, length of oblique lines = 53′). Cubes and Rectangles contained the lines from the Lines condition, and the Cubes condition contained the lines from the Rectangles condition.

### Procedure

Observers were instructed to fixate a central fixation dot during each trial. After stimulus presentation, the screen remained blank for a maximum period of 3 s, during which the observer was required to make a response by pressing one of two handheld push buttons. The task was to indicate whether the bottom line of the vernier was offset to the left or right, relative to the top line of the vernier. Auditory feedback was provided after incorrect or omitted responses. The vernier offset was controlled through the adaptive staircase procedure PEST (Taylor & Creelman, 1967), but thresholds (75% correct criterion) were determined from the post hoc fit of the psychometric function (cumulative Gaussian) to the log-scaled data. In order to avoid extremely large vernier offsets, we restricted the PEST procedure to not exceed 1,600′′ (arcsec) (i.e., twice the starting value of 800′′). The offset directions (left or right) were randomized, but no more than four consecutive trials could have the same direction. Each condition was presented in separate blocks of 80 trials. All conditions were measured twice (i.e., 160 trials) and randomized individually for each observer. To compensate for possible learning effects, the order of conditions was reversed after each condition had been measured once.

### Model simulations

The same stimuli were presented to the LAMINART model of visual perception, which previously explained a variety of uncrowding effects (Francis et al., 2017; Manassi et al., 2015). To make this study self-contained, we summarize the key model properties that are necessary for the current discussion. The model emulates aspects of visual cortical processing, using integrate-and-fire model neurons, which establishes the time scale of visual processing in the model (and determines how quickly visual information fades away from the neural circuit). The model circuits combine bottom-up information from a stimulus with top-down control that selects and segments image information in order to best achieve certain tasks. For the vernier discrimination task, the top-down goal of the model is to segment the visual representations of the vernier from the flanking elements. In this way, the model can avoid interference effects (crowding) that the flankers might have on the templates used to evaluate the direction of vernier offset based on the visual information in the baseline segmentation layer. One template sums neural activity across the spatial regions that indicate a left-shifted template to produce a left-template score, *S_L_*. Another template sums neural activity across the spatial regions that indicate a right template to produce a right-template score, *S_R_*. Consistent with many theories of crowding, these templates are large enough to cover the vernier stimulus and nearby flanking elements. The model computes a contrast score for a right-shifted vernier (all model stimuli were right-shifted verniers):

$$\begin{array}{c} C=\frac{S_{R}-S_{L}}{100+S_{R}+S_{L}} \end{array}$$

Crowding occurs when the flankers contribute to the templates, thereby reducing the relative difference between the left and right templates. This contrast calculation is performed for each 20-ms time step. Model evidence is the sum of the contrast values that maximized model performance (generally summing values from 20 to 100 ms after vernier onset). Larger model evidence values correspond to better performance (e.g., smaller thresholds) on a vernier discrimination task. Since our empirical measurements estimate thresholds for performance, we plot model evidence values with an inverted y-axis. If the segmentation process can move the flanker elements to a separate segmentation layer from the vernier, then they hardly enter the model evidence calculation, which will lead to good performance.

The simulations in Francis et al. (2017) heavily depended on the formation of connections between disparate elements (which roughly corresponded to grouping), but these connections hardly matter for the current stimuli because the flankers are almost all fully connected. For this reason, we used an updated version of the model (Kon & Francis, 2022) that allows top-down control to prevent the formation of connections. Most of the explanatory power of the model for the current simulations comes from the segmentation process. To simplify the simulations, the model used a fixed location for the segmentation signals that led to good segmentation when it was feasible (e.g., the flankers are large enough to be selected and separated from the vernier lines). Adding a bit of noise to the location of the segmentation signal would hardly make any difference for these stimuli (see Francis et al., 2017). A more detailed explanation of the model dynamics, along with examples of stimuli used in the experiments, is provided below.

## Results

### Experiment 1

Previous studies have shown that crowding is strongly determined by spatial grouping: When flanking elements group together, the vernier is released from crowding (i.e., uncrowding effect; Francis et al., 2017; Manassi et al., 2012; Manassi et al., 2013). In Experiment 1, we investigated the role of stimulus duration in spatial grouping.

### Methods

In Experiment 1, we investigated the temporal dynamics of uncrowding for three different flanking configurations (the same shown in Figure 1). Participants discriminated the left or right offset of a vernier either alone or flanked by Lines, Rectangles, or Cubes for 20 or 160 ms of total stimulus duration.

### Behavioral results

**Figure 2.**
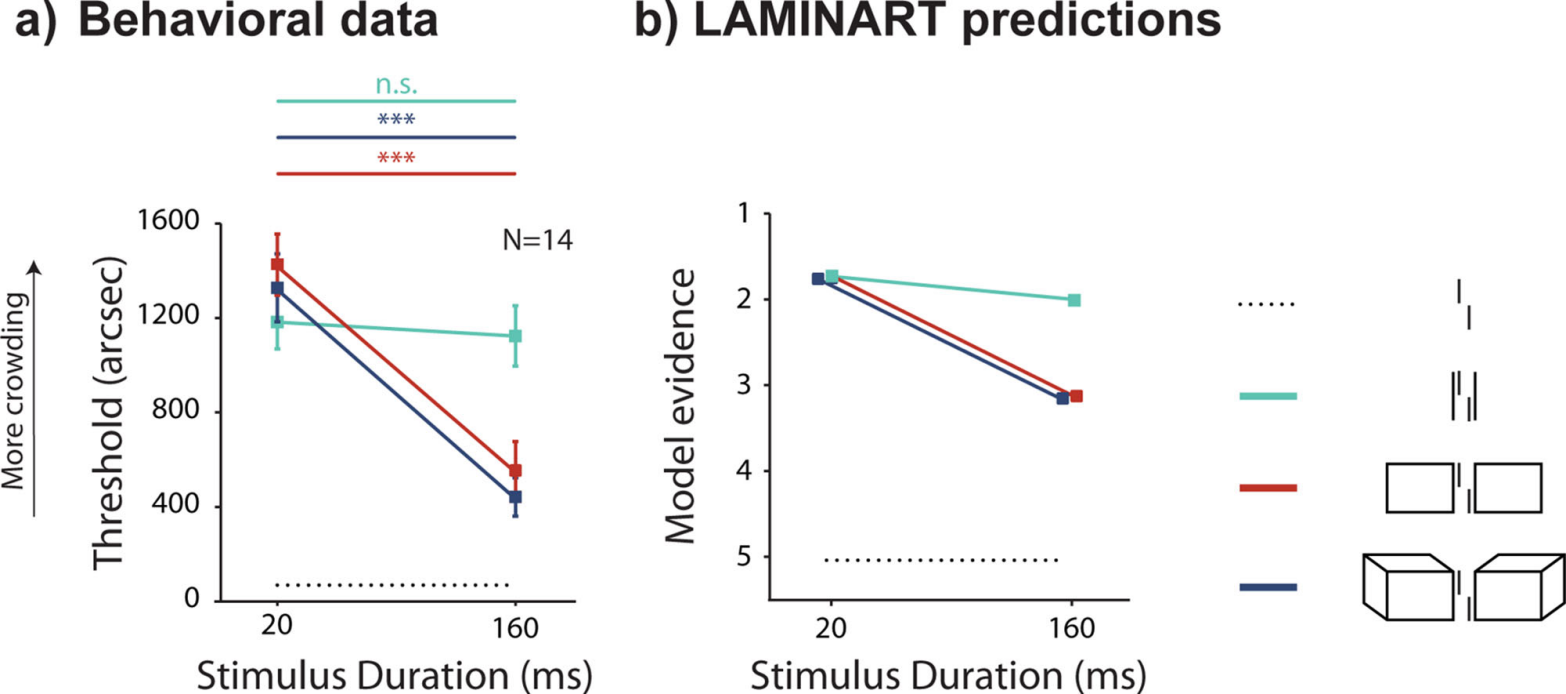
Experiment 1: Temporal dynamics of uncrowding. Results from empirical data (**a**) and simulations from the model (**b**). Mean vernier offset discrimination thresholds as a function of stimulus duration for the three flanker types: Lines, Rectangles, and Cubes. The black dotted line indicates the threshold for an unflanked vernier displayed for 20 ms (extended through the entire graph as reference). Error bars indicate the standard error of the mean. Straight lines on top of graph indicate the results of post hoc paired *t*-tests between the two stimulus durations (20 and 160 ms), for each flanker type (different colors). When the vernier was flanked by Lines (green line), thresholds remained at a high constant level at both stimulus durations. When the vernier was flanked by Rectangles (red line) or Cubes (blue line), thresholds strongly decreased for a stimulus duration of 160 ms. The model's simulations reflect the pattern observed in the behavioral data (the y-axis is reversed since high model evidence corresponds to a low threshold). Lines indicating performance for flanking Rectangles and Cubes overlap and have the same trend.

When vernier and flankers were presented for 160 ms, vernier discrimination thresholds increased compared to the unflanked condition (Figure 2; pairwise one-tailed *t* test with Bonferroni correction, adjusted *p* values: vernier alone vs. vernier flanked by Lines, 160 ms, *t*(13) = −8.22,  *d* = 2.20, *p* < 0.001; Rectangles, 160 ms, *t*(13) = −3.96,  *d* = 1.06,  *p* < 0.01; Cubes, 160 ms, *t*(13) = −4.59,  *d* = 1.23,  *p* < 0.01). We found a significant interaction between stimulus duration and flanker type (two-way repeated-measures analysis of variance [ANOVA]: *F*(2,  26) = 12.0, partial η^2^ = 0.48, *p* < 0.001). Crowding was lower for the Cubes or Rectangles conditions compared to Lines (post hoc pairwise Bonferroni-corrected comparisons; we report the adjusted *p* values): Lines 160 ms vs. Cubes 160 ms, t(51.8) = 4.81,  *d*  =  1.28, *p* < 0.001; Lines 160 ms vs. Rectangles 160 ms, *t*(51.8) = 4.04,  *d*  =  1.08, *p* < 0.01; and Cubes 160 ms vs. Rectangles 160 ms, *t*(51.8) = −0.79, *d*  =  0.21, *p* = 1.0.

These results are in line with extensive literature on uncrowding (Bornet et al., 2021; Bornet et al., 2021; Choung et al., 2021; Choung et al., 2023; Francis et al., 2017; Herzog, 2022; Herzog et al., 2015; Herzog & Manassi, 2015; Jastrzębowska et al., 2021; Manassi et al., 2012; Manassi et al., 2013; Manassi et al., 2015; Manassi et al., 2016; Saarela et al., 2009; Saarela et al., 2010; Sayim et al., 2010; Schwetlick, Manassi, Herzog, & Francis, 2025; Tiurina et al., 2022).

Interestingly, when vernier and flankers were presented for 20 ms, strong crowding occurred in all conditions (Figure 2a; vernier alone vs. vernier flanked by Lines:  *t*(13) = −9.71,  *d*  =  2.59, *p* < 0.001; Rectangles: *t*(13) = −8.77,  *d*  =  2.34,  *p* < 0.001; Cubes: *t*(13) = −10.47,  *d*  =  2.80,  *p* < 0.001). We found no significant difference in crowding strength between Lines, Rectangles, and Cubes configurations when presented for 20 ms (Lines 20 ms vs. Cubes 20 ms, (51.8) = −1.02,  *d*  =  0.27, *p* = 1.0; Lines 20 ms vs. Rectangles 20 ms, *t*(51.8) = −1.72,  *d*  =  0.46, *p* = 1.0; Cubes 20 ms vs. Rectangles 20 ms, *t*(51.8) = −0.69, *d*  =  0.18, p = 1.0). We propose that grouping of the flankers does not occur for a shorter stimulus duration, and hence, crowding remains strong in all conditions. For longer stimulus durations, there is enough time to initiate grouping of the flankers away from the target, and thus uncrowding occurs (20 ms vs. 160 ms; Cubes, *t*(37.2) = 5.95, *d*  =  1.59, *p* < 0.001; Rectangles, *t*(37.2) = 5.86, *d*  =  1.57, *p* < 0.001; Lines, *t*(37.2) = 0.40, *d*  =  0.10,  *p* = 1.0; see also Doerig, Schmittwilken, Sayim, Manassi, & Herzog, 2020, Figure 3).

**Figure 3.**
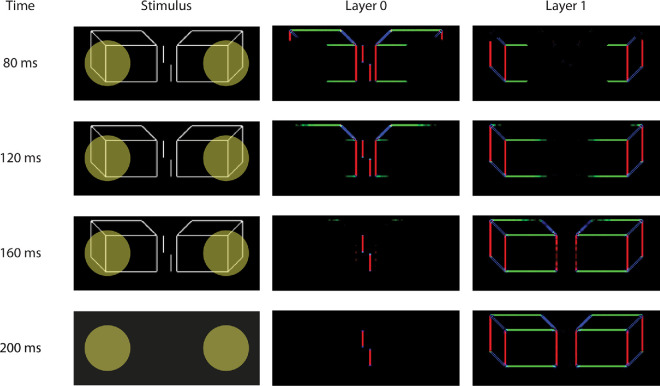
Model simulations for the flanking Cubes condition. The top-down selection signals (yellow circles) spread across the connected contours of the flankers and shift the flanker representation to Layer 1. The vernier is left behind in Layer 0, which makes it easy to identify the vernier shift. A colored pixel indicates the presence of an oriented contour. The colors of the pixels (red for vertical, green for horizontal, and blue for diagonals) are used for visualization purposes only and do not reflect differences in model processing.

### Model simulations

The LAMINART model qualitatively reproduces our results (Figure 2b). To understand how these results come about, Figure 3 shows the LAMINART model's response to the flanking Cubes when the stimulus is presented for 160 ms. The model has two separate segmentation layers, which we call Layer 0 and Layer 1. Each segmentation layer represents the oriented contours of the stimulus image. Here, vertical contours are indicated by red pixels, horizontal contours are indicated by green pixels, and two diagonal orientations are indicated by blue pixels. Each pixel color represents a specific contour orientation for visualization purposes. At 80 ms after stimulus presentation, most of the contours are represented most strongly in Layer 0. However, a dynamic transfer of information about the flankers from Layer 0 to Layer 1 is ongoing because of top-down selection signals (indicated by the yellow circles on the stimulus). These selection signals spread across the locations of connected contours and shift the contours in Layer 0 to the same corresponding pixel locations in Layer 1. This selection/segmentation process is evident as the simulation time progresses, and by around 160 ms, the contours corresponding to the vernier are almost isolated in Layer 0, while the contours corresponding to the flanker are almost entirely represented in Layer 1. The neural circuits that support the segmentation process are described in Francis et al. (2017). A longer stimulus duration allows the selection signal to spread along the borders of the Rectangles and Cubes, identifying them as separate objects and segregating them into different layers. However, for very short-duration stimuli, the selection/segmentation process does not have sufficient time to segment the representations of the flankers and the vernier, so crowding is strong.

When the segmentation has separated the representation of the flankers from the representation of the vernier, templates that calculate evidence for a right-shifted versus a left-shifted vernier are quite different, meaning the task is relatively easy (a small shift is sufficient for good detection, so thresholds will be small; see Figure 2).

**Figure 4.**
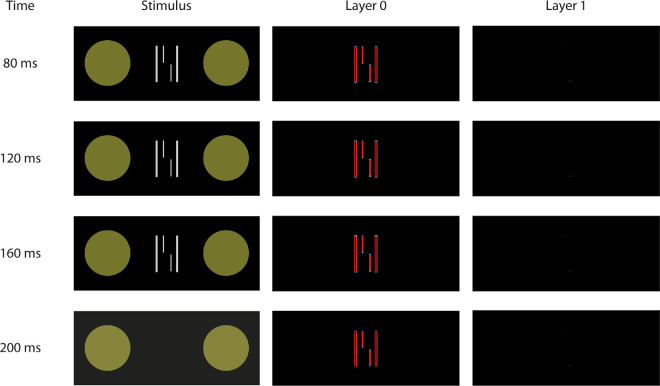
Model simulations for the flanking Lines condition. The top-down selection signals (yellow circles) miss the small line flankers and do not promote segmentation of the vernier and flankers. Crowding makes it difficult to identify the vernier shift. A colored pixel indicates the presence of an oriented contour. The colors of the pixels (red for vertical, green for horizontal, and blue for diagonals) are used for visualization purposes only and do not reflect differences in model processing.

The model behavior is quite different for the Lines stimulus shown in Figure 4. The flanker lines are very close to the vernier, so it is difficult for the selection signals to cover only the flanking elements without also covering some of the vernier. In the simulation shown in Figure 4, the flanking Lines are not segmented, so they interfere with the template calculations, meaning the task is difficult (thresholds will be large). In other simulations, the selection signals might cover both the target and the flankers, in which case the contours for all elements are transferred to Layer 1. This segmentation does not help because the template calculations in Layer 1 will suffer from the same kind of interference (crowding; see Figure 2). A longer stimulus duration does not bring any advantage in this case. So, the key difference between the Cubes and Lines stimulus is that the flankers in the Cubes condition provide a larger target for the top-down selection signals. In follow-up simulations, we explore the dynamics of the selection/segmentation process and see that it provides a consistent explanation of the empirical data.

Taken together, these results show that crowding strength can be modulated by stimulus duration when the flankers can be grouped (segmented) away from the vernier. Hence, grouping the flankers away from the target is a time-consuming process that does not occur with very short stimulus durations.

### Experiment 2

In Experiment 1, we showed that grouping requires a minimum stimulus duration. With a 20-ms stimulus duration, the vernier was equally crowded by Lines, Rectangles, and Cubes. With a 160-ms stimulus duration, the Rectangles and Cubes grouped away from the target, releasing the vernier from crowding. This result aligns with previous findings (Doerig et al., 2020) and was well predicted by the LAMINART model. As this grouping process is time-dependent, we investigated whether a brief preview of the flankers could initiate their grouping away from the target, thereby reducing crowding for a vernier presented much later.

#### Methods

**Figure 5.**
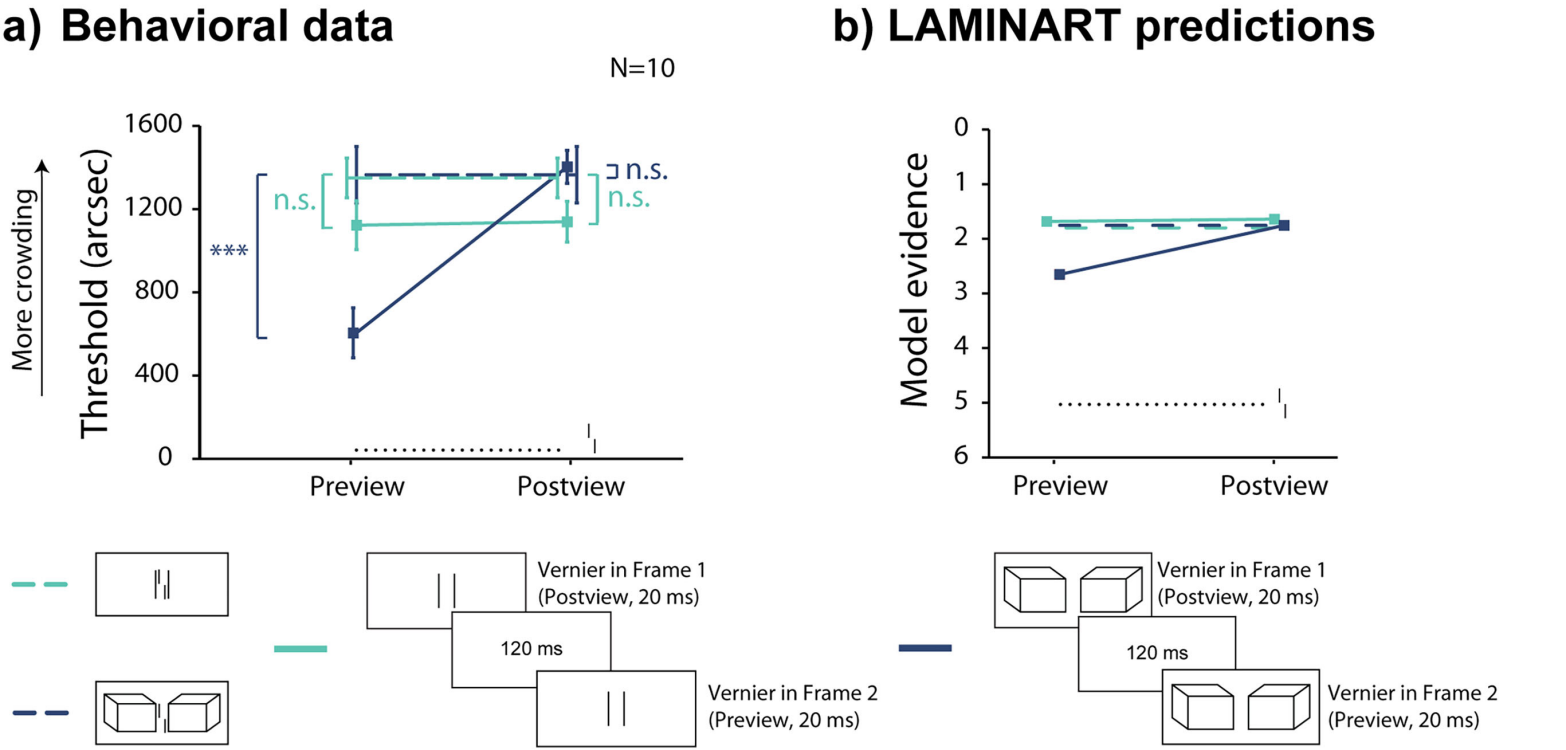
Experiment 2: Uncrowding in time. Results from empirical data (**a**) and simulations from the model (**b**). Mean vernier offset discrimination thresholds for preview/postview conditions for Cubes (in blue) and Lines (in green). The black dotted line indicates the threshold for an unflanked vernier displayed for 20 ms (extended through the entire graph as reference). Colored dashed lines indicate baseline crowding conditions for each flanker type, extended through the entire graph as reference. Error bars are standard error of the mean. Asterisks indicate a significant difference with baseline (dashed lines, crowding condition of vernier flanked by Cubes or lines for 20 ms) in post hoc paired *t*-tests. A preview of the Cubes induced a release from crowding (blue line). A preview of the Lines did not change crowding strength (green line). The model's simulations (**b**) reflect the pattern observed in the behavioral data (the y-axis is reversed since high model evidence reflects good performance).

In Experiment 2, we measured vernier offset discrimination thresholds for a vernier flanked by either Lines or Cubes, which was either preceded (preview) or followed (postview) by a 120-ms interstimulus interval (ISI) and then a 20-ms display containing only the flankers (Figure 5). Due to the similarity in performance between the Rectangles and Cubes conditions (Figure 2), we only included Cubes in the following experiments. We hypothesized that uncrowding in time occurs when previewing flankers with the potential for grouping (i.e., Cubes), whereas crowding remains strong when previewing flanking Lines that do not lead to uncrowding at longer stimulus durations. We also hypothesized that a postview condition (i.e., presenting Lines and Cubes after the vernier) would not lead to uncrowding.

#### Behavioral results

As shown in Experiment 1, displaying the vernier flanked by Lines or Cubes for 20 ms led to strong crowding (pairwise one-tailed *t*-test with Bonferroni correction: vernier alone vs. vernier flanked by Lines: t(9) = −12.13, *d*  =  3.83,  *p* < 0.001, Cubes: t(9) = −9.98,  *d*  =  3.15,  *p* < 0.001), and we found a significant interaction between flanker type and design condition (two-way repeated-measures ANOVA: *F*(2,  18) = 25.26, partial η^2^ = 0.63, *p* < 0.001). Giving a preview of the flanking Cubes reduced crowding compared to displaying the vernier flanked by Cubes for 20 ms (Figure 5a; post hoc pairwise Bonferroni-corrected comparisons [we report the adjusted *p*-values]: Cubes 20-ms duration vs. Cubes preview, *t*(35.9) = 7.44, *d*  =  2.35, *p* < 0.001). Notably, the improvement in performance is comparable to displaying the vernier and the flankers for longer durations (see Figure 2a in Experiment 1), despite the target being on the screen for only 20 ms. The preview of the Lines did not reduce crowding strength (Figure 5a; Lines 20-ms duration vs. Lines preview, *t*(35.9) = 1.62, *d*  =  0.51, *p* = 1.0). Displaying the flankers after the flanked target (postview condition) also did not affect crowding strength (Figure 5a), regardless of flanker type (Cubes 20-ms duration vs. Cubes postview, *t*(35.9) = −0.37, *d*  =  0.12,  *p* = 1.0; Lines 20-ms duration vs. Lines postview, *t*(35.9) = −1.09, *d*  =  0.34, *p* = 1.0).

#### Model simulations

The simulations from the LAMINART model align with the behavioral data (Figure 5b). When the Cubes are presented in isolation, the selection/segmentation process begins. The contour representations persist through the 120-ms ISI (Francis, Grossberg, & Mingolla, 1994), so there is sufficient time for the model to segment most of the contours of the Cubes. When the vernier appears, it is represented in Layer 0 by itself, thereby leading to uncrowding. The same segmentation advantage does not happen for Lines because the selection signals usually either cover the lines and the space where the vernier will (eventually) appear or do not cover the flanking Lines at all. Consequently, the vernier and flanker representations remain in a common segmentation layer, and crowding is strong. The postview conditions provide no advantage for the model because they do not enable segmentation of the flanker and vernier representations. Instead, the vernier representation largely fades away during the ISI, and any subsequent segmentation of the flankers has no impact on the vernier template calculations.

Taken together, these results provide evidence for a novel preview effect that, unlike previously reported preview effects in the literature (Chung, 2016; Chung & Patel, 2022; Greenwood, Sayim, & Cavanagh, 2014; Huckauf & Heller, 2004; Scolari, Kohnen, Barton, & Awh, 2007; Soo, Chakravarthi, & Andersen, 2018), occurs only when flankers have the potential to be grouped away from the target for longer stimulus durations.

### Experiment 3

Following the demonstration of this configuration-specific preview effect (Figure 5), we explored its temporal dynamics by investigating how stable the effect would be for longer ISIs.

#### Methods

**Figure 6.**
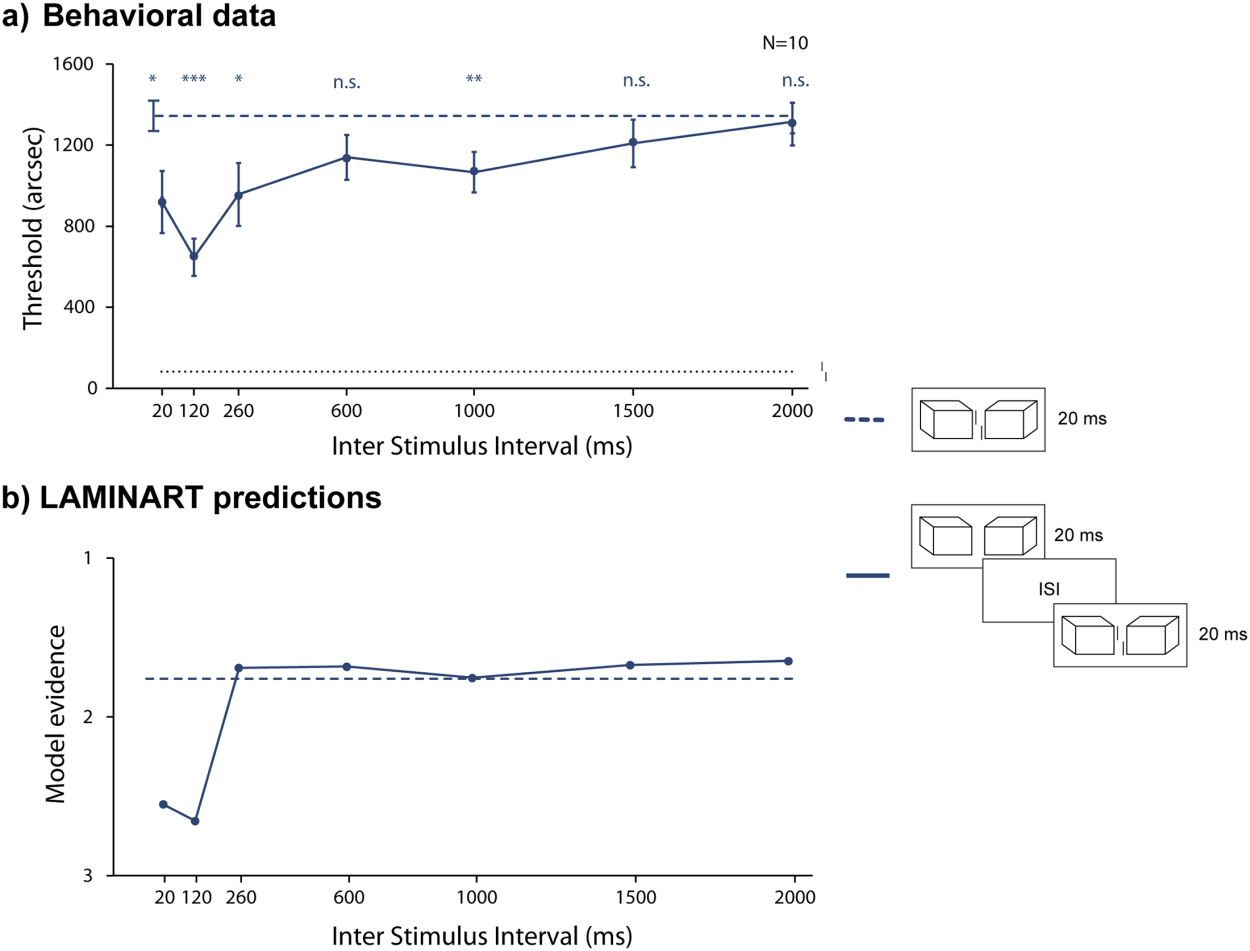
Experiment 3: The preview effect is long-lasting. Results from empirical data (**a**) and simulations from the model (**b**). Mean vernier offset discrimination thresholds as a function of ISI duration. The black dotted line indicates the threshold for an unflanked vernier displayed for 20 ms (extended through the entire graph as reference). The blue dashed line indicates the vernier flanked by Cubes with a 20-ms stimulus duration. Error bars indicate the standard error of the mean. Asterisks on top of bars indicate a significant difference with baseline (blue dashed line, crowding condition of vernier flanked by Cubes for 20 ms) in post hoc paired *t*-tests. Crowding decreased for ISIs of up to 1,000 ms. The model simulations reflect the pattern observed in the behavioral data (the y-axis is reversed since high model evidence corresponds to low thresholds), although the preview advantage only exists for shorter ISIs.

In Experiment 3, we measured vernier offset discrimination thresholds by displaying the Cubes alone, followed by a variable ISI, and then by the display with Cubes and the vernier (Figure 6). We tested ISIs ranging from 20 to 2,000 ms.

#### Behavioral results

There was a significant effect of ISI (one-way repeated measures ANOVA: *F*(7,  64) =  4.03, partial η^2^ =  0.31,  *p*  =  0.001). Providing a preview of the flanking Cubes led to uncrowding for some ISIs up to 1,000 ms (Figure 6a**,** post hoc one-tailed pairwise *t*-tests after false discovery rate [FDR] correction [we report the adjusted *p*-values]: 20 ms, *t*(64) = 2.6, *d* = 0.82,  *p* = 0.03; 120 ms, *t*(64) = 6.8,  *d* = 2.15,  *p* < 0.001; 260 ms, *t*(64) = 2.3, *d* = 0.73,  *p* = 0.04; 600 ms, *t*(64) = 2.0, *d* = 0.63,  *p* = 0.05; 1,000 ms, *t*(64) =  3.8, *d* = 1.20,  *p* < 0.01; 1,500 ms, *t*(64) = 1.4,  *d* = 0.44,  *p* = 0.12; 2,000 ms, *t*(64) = 0.3, *d* = 0.09,  *p* = 0.39).

An observed empirical improvement at an ISI of 20 ms compared to the no-preview condition appears noteworthy, considering its brevity, which would typically not allow sufficient time for segmentation to manifest, as evidenced in Experiment 1. This result may be due to a flickering phenomenon: Crowding decreases when the target “blinks” (disappears and then reappears) from the display with the flankers for 20 ms, or its onset is delayed by 210 ms with respect to the flankers (Greenwood et al., 2014). Nevertheless, it is important to note that a general flickering effect cannot account for the whole body of results, given that the release from crowding occurs for Cubes but not for Lines (Figure 5). Hence, our preview effect is flankers specific, and thus it cannot be due to a mere flickering effect.

#### Model simulations

The model shows similar behavior (Pearson correlation coefficient between model evidence and behavioral thresholds is –0.83; Figure 6b), with the preview effect giving small improvements in performance for up to a 120-ms ISI. However, the model does not account for uncrowding for longer ISIs (i.e., 260 ms, 600 ms, 1,000 ms). When presenting Cubes in isolation, they easily migrate to their designated segmentation layer, thereby allowing the vernier to stand out more easily when the second frame is presented. In human observers, this process unfolds over a longer time scale than that employed by the model. For ISIs over 120 ms, the representation of the segmented Cubes fades and cannot be integrated with the frame of Cubes and vernier, and model evidence is low. The model, therefore, captures some aspects of, but falls short of completely matching, the behavioral data.

### Experiment 4

**Figure 7.**
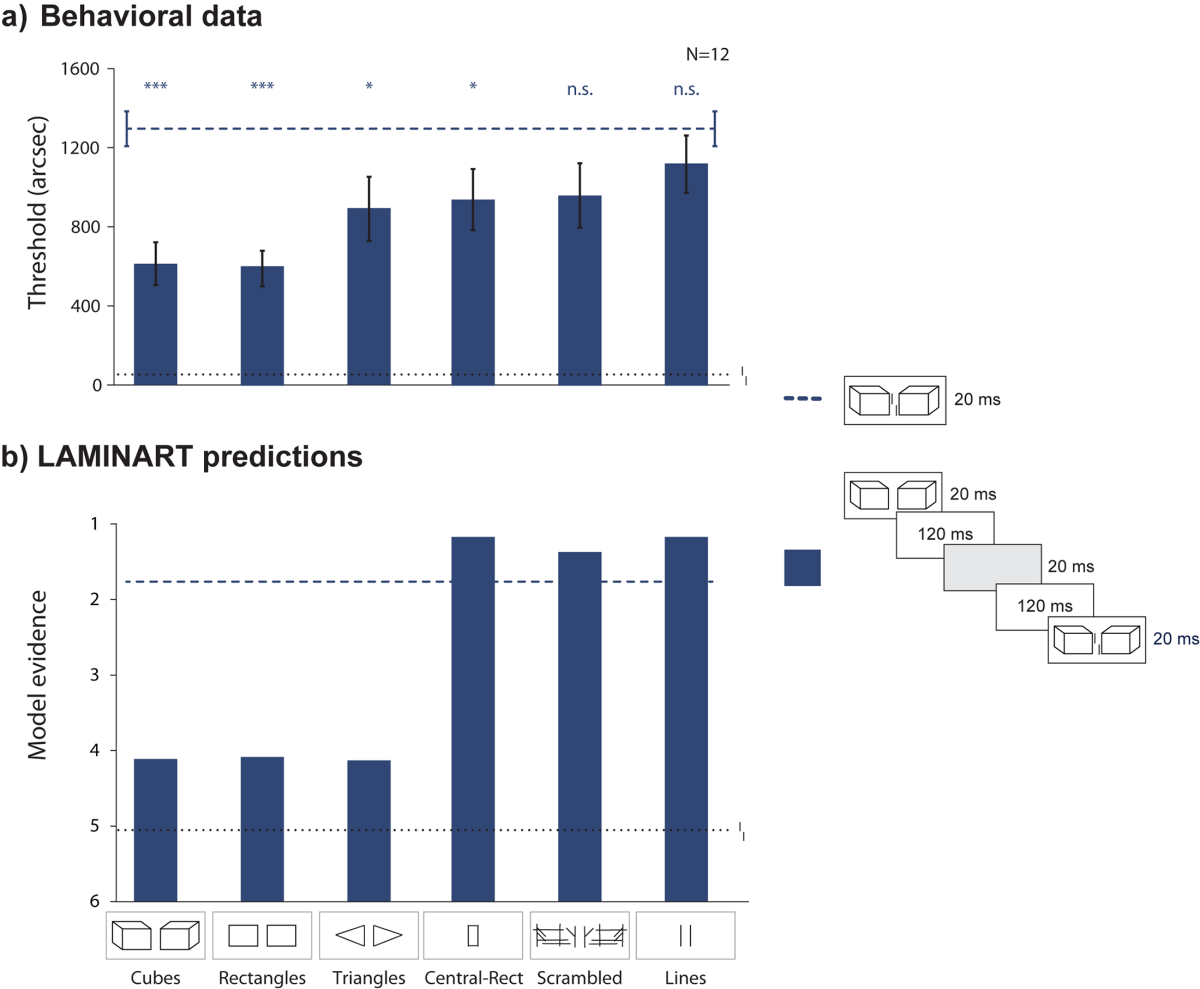
Experiment 4: The preview effect is gated by grouping processes occurring over time. Results from empirical data (**a**) and simulations from the model (**b**). Mean vernier offset discrimination thresholds as a function of the type of flanker displayed in the intermediate frame. The black dotted line indicates the threshold for an unflanked vernier displayed for 20 ms (extended through the entire graph as reference). The blue dashed line indicates the baseline crowding condition: vernier flanked by Cubes, 20-ms stimulus duration. Error bars indicate the standard error of the mean. Asterisks on top of bars indicate a significant difference from the baseline (blue dashed line). The model's simulations mostly reflect the pattern observed in the behavioral data (the y-axis is reversed since high model evidence reflects good performance).

To delve deeper into the underlying dynamics of this uncrowding effect, we inserted an intermediate frame of flankers in Experiment 4, between the initial presentation of Cubes alone and the subsequent presentation of the Cubes with the vernier (Figure 7). Possible intermediate displays were Cubes, Rectangles, Triangles, Central Square, Scrambled Cubes, and Lines. If uncrowding occurs due to grouping processes unfolding over time, we hypothesized that inserting an intermediate frame between the preview and the crowded stimuli would lead to uncrowding depending on the nature of the intermediate flankers. As Cubes exactly match the preview stimuli, Rectangles preserve most of their structural features, and Triangles form closed‐contour Gestalt objects, we predicted that the flanker grouping process should propagate through these types of frames and ultimately allow the vernier in the final frame to be uncrowded. The Central-Rectangle condition was more exploratory: Although it also qualifies as a coherent Gestalt, its single, centrally located shape (rather than two symmetric flankers beside the vernier) differs strikingly from the preview configuration. This contrast allowed us to probe whether the visual system favors spatial similarity (i.e., matching lines in the same positions) or the more abstract principle of perceptual grouping. By contrast, line segments and Scrambled Cubes—both lacking an inherently groupable structure—were expected to crowd.

#### Methods

In addition to the initial frame containing the Cubes alone, we presented the three flanking configurations used in previous designs (Lines, Rectangles, and Cubes; Figure 1), along with three novel ones: Scrambled Cubes, Triangles, and Central-Rectangle (Figure 7). Scrambled Cubes were obtained by shuffling the lines from the Cubes condition. The Triangles’ inner sides were 84′, while the oblique sides were 124.3′ long. The Central-Rectangle had the same height as the other flankers (84′) and a width of 46′ (as the spacing between the Lines).

In each trial, we presented the Cubes alone for 20 ms, followed by an ISI of 120 ms, one of the aforementioned flankers alone for 20 ms, another ISI of 120 ms, and then the vernier flanked by the Cubes for 20 ms.

#### Behavioral results

In the behavioral data (Figure 7a), there was a significant effect of flanker type (one-way repeated-measures ANOVA: *F*(6,  66) = 11.33,  partial η^2^ = 0.51,  *p* < 0.001). As expected, crowding decreased when presenting Cubes (post hoc pairwise comparisons with FDR correction [we report the adjusted *p*-values]: Cubes *t*(11) = 6.43, *d* = 1.86,  *p* < 0.001) or Rectangles *t*(11) = 11.46,  *d* = 3.31,  *p* < 0.001) as intermediate frames, since they share the same global characteristics. Following the introduction of intermediate presentations of good Gestalt configurations, such as Triangles (*t*(11) = 3.46,  *d* = 1.0,  *p* = 0.02) and a Central-Rectangle (*t*(11) = 3.11, *d* = 0.89,  *p* = 0.02), we still found uncrowding. This last result is particularly compelling because it demonstrates that the visual system prioritizes perceptual grouping over local feature similarity. Importantly, uncrowding did not occur with Scrambled Cubes (*t*(11) = 2.60,  *d* = 0.75,  *p* = 0.05) or Lines (*t*(11) = 1.60,  *d* = 0.46,  *p* = 0.18) as intermediate frames, indicating that these distractors disrupt the grouping-in-time process. Comparisons from the post hoc analysis are shown in Table 1. Taken together, these results showed that the preview effect occurred only with good Gestalt configurations as intermediate frames, providing evidence that the preview effect is gated by grouping processes over time.

**Table 1.** Results of the post hoc pairwise comparisons of Experiment 4. The *p*-values, FDR-corrected (lower triangle), and test statistics *t*(11) (upper triangle) are shown for each stimulus combination. Abbreviations in the table: Cub20 = Cubes displayed with the vernier for 20 ms; other conditions had flankers displayed in the intermediate frame.

	Cub20	Cubes	Rectangles	Triangles	Central-Rect	Scrambled	Lines
Cub20	—	6.43	−11.46	−3.46	−3.11	−2.60	−1.60
Cubes	<0.001	—	−0.29	1.85	2.12	2.98	4.70
Rectangles	<0.001	0.82	—	3.13	3.76	3.75	−5.86
Triangles	0.02	0.14	0.02	—	−0.75	−0.89	−2.06
Central-Rect	0.02	0.10	0.01	0.52	—	−0.20	−1.78
Scrambled	0.05	0.03	0.01	0.46	0.84	—	−1.57
Lines	0.18	<0.01	<0.001	0.10	0.14	0.18	—

#### Model simulations

Figure 7b shows that the model simulations explain most of the empirical data (Pearson correlation coefficient between model evidence and behavioral thresholds is –0.81). The model's explanation is much the same as for the results in Figure 5b. The selection signal partially spreads across the contours of the Cubes during the original presentation and the subsequent ISI. At the presentation of the intermediate flankers, there is pixel overlap between the selected contours and the contours of the new flanker. As a result, the selection process continues with the contours of the new flanker elements. This process does not occur for the Lines as intermediate flankers because the selection signal tends to be placed far from the location of the lines (to avoid selection of the subsequent vernier). Oftentimes, the selection signal has not spread to the location of the lines, and so the selection signal gets lost during the ISI after the intermediate flankers. The same kind of effect happens for the Scrambled Cubes because the innermost lines of that flanker are largely disconnected from the farther away scrambled lines. For both of these intermediate flankers, some trials do produce a preview effect because the selection signal is placed close enough to the innermost lines that the selection signal spreads to them during the first ISI.

The main qualitative discrepancy between the model and the empirical data is for the central rectangle intermediate flanker condition (Figure 7, Central-Rectangle). The model treats this stimulus much like the intermediate Lines condition, but the top and bottom of the central rectangle cause some additional crowding that makes the model’s predicted performance a bit worse than the Lines condition. In contrast, the empirical data show a modest preview effect.

Another discrepancy between the model and the behavioral data is represented by the Triangles. In the behavioral data (Figure 7a, Table 1), Triangles are significantly different from Rectangles (*t*(11) = 3.13,  *d* = 0.90,  *p* = 0.02) but not from Cubes (*t*(11) = 1.85,  *d* = 0.53,  *p* = 0.14). In the model, the three conditions give comparable uncrowding. Although the nonsignificant difference between Triangles and Cubes in the behavioral data may simply reflect limited statistical power, the model treats these two conditions as nearly identical because the triangle's apex extends to the far vertical edge of the preview cube's front face. During the preview, selection signals propagate along that contour; when the triangle appears, its tip seamlessly integrates into the existing activation. The same occurs for both Cubes and Rectangles, but not for the Central-Rectangle or for Lines. In the Scrambled Cubes condition, the inner lines remain too disconnected from the preview cube's boundary to capture any residual signal.

## General discussion

Our results provide evidence for a novel preview effect in visual crowding: A very brief flankers presentation (20 ms) can lead to uncrowding of subsequently presented objects. This effect (Figure 5) occurs only if the flankers have the potential to group (Experiment 3), it occurs for up to 1 second, and it is governed by time-dependent grouping processes (Experiments 2–4).

For 20-ms presentations, crowding is strong for all flanker configurations. (Experiment 1; Doerig et al., 2020). For Cubes, uncrowding requires stimulus durations of at least 160 ms. For these longer stimulus durations, we propose that the brain has sufficient time to group the flankers away from the vernier target, therefore reducing crowding. The same result can be obtained with a preview of the flankers, even if presented for just 20 ms (Experiment 2). We propose that displaying the flanking Cubes alone (i.e., without the vernier target) initiates processing of the Cubes, which continues during the ISI. The full representations can then be used to segment the vernier away. Hence, spatiotemporal grouping is key in crowding (Bornet et al., 2021; Bornet et al., 2021; Choung et al., 2021; Choung et al., 2023; Francis et al., 2017; Herzog, 2022b; Herzog et al., 2015; Herzog & Manassi, 2015; Jastrzębowska et al., 2021; Manassi et al., 2012; Manassi et al., 2013; Manassi et al., 2015; Manassi et al., 2016; Saarela et al., 2009; Saarela et al., 2010; Sayim et al., 2010; Schwetlick et al., 2025; Tiurina et al., 2022). Notably, the total stimulus duration of the Cubes is 20 ms for the preview and 20 ms for their presentation together (i.e., 40 ms altogether), which is much shorter than the critical duration of 160 ms. Hence, what matters is not the stimulus duration *per se* but the processing time. Our results challenge the idea that the initial stages of visual processing in a feedforward fashion are most essential for perceiving stimuli under crowding conditions. Instead, crowding seems to be a dynamic process fundamentally shaped by grouping mechanisms that unfold over time.

A large part of our empirical results is well explained by the recurrent segmentation processes in the LAMINART model (Francis et al., 2017; Manassi et al., 2015). Uncrowding in time occurs when the flanking elements are segmented away from the target vernier. Recurrent connections allow the efficient spreading of selection signals across layers and ultimately segmentation of the different objects (see also Wallis et al., 2019). The model's segmentation process plays the role of grouping the flanking elements.

Our preview effect led to uncrowding of the subsequently presented crowded vernier, even if presented 1 s before (Experiment 3). Thus, their representations must be held in visual memory for up to a second, similarly to the time scales of iconic memory (Phillips, 1974; Sperling, 1960). It is important to mention that 1 s is a relatively long time in visual processing, especially considering that object recognition can occur much faster (i.e., even within 300 ms; Kirchner & Thorpe, 2006; Rousselet, Macé, & Fabre-Thorpe, 2004; Thorpe et al., 1996; VanRullen & Thorpe, 2001). We suggest that in easy recognition tasks, the detection of features may be sufficient for discrimination, such as distinguishing animals from cars. This is similar to deep neural networks, which perform at the human level in animal detection tasks based on features like fur. However, neural networks struggle when texture information is not available and proper segmentation is required (Ballester & Araujo, 2016; Brendel & Bethge, 2019; Geirhos et al., 2018).

Object segmentation is challenging because the number of possible groupings increases exponentially with the amount of information, making it computationally infeasible even with relatively simple elements (Comaniciu, Meer, & Member, 2002; Felzenszwalb & Huttenlocher, 2004; Ren & Malik, 2003; Shi & Malik, 2000). Feedback connections might be crucial for achieving such computations (Chicherov, Plomp, & Herzog, 2014; Jastrzębowska et al., 2021). In light of this, we suggest that the brain seizes any opportunity to group elements when possible. For the flanking Cubes, grouping is feasible since they can be identified as individual objects. The preview facilitates recognition and segmentation of the Cubes, while the Lines are always grouped together with the vernier. Uncrowding does not occur when the flankers are shown after the vernier (postview condition; Figure 5) because, during short presentations, only one object can be identified.

As long as there is no disruptive information between the Cubes and the vernier, the grouping process continues (Experiment 4). Interestingly, these segmentation processes extend over several hundred milliseconds, suggesting the involvement of feedback connections from higher visual areas. Recent neuroimaging studies in primates (Kim & Pasupathy, 2024) support our hypothesis by highlighting the role of global flanker configuration in crowding.

Our results differ from standard priming effects, where providing a cue about the target for brief durations before the trial leads to increased access to its representation at that spatial location, resulting in faster or more accurate responses (Hilchey, Leber, & Pratt, 2018; Janiszewski & Wyer, 2014; Kristjánsson, Wang, & Nakayama, 2002; Kristjánsson & Campana, 2010; Kristjánsson & Driver, 2008; Theeuwes, 2013; Wilkinson, Wilson, & Ellemberg, 1997; Yeshurun & Rashal, 2010). This standard priming effect cannot explain our results, as there is no target uncertainty in our case. The spatial location and main characteristics of the target are constant across trials, so priming should also occur in the flanking Lines conditions. Similarly, our results cannot be explained by standard preview effects in visual crowding. Previous studies have shown that presenting the flankers before a crowded stimulus can result in reduced crowding (Chung, 2016; Chung & Patel, 2022; Greenwood et al., 2014; Harrison & Bex, 2014; Huckauf & Heller, 2004; Scolari et al., 2007; Soo et al., 2018). However, in our case, the preview effect is strictly dependent on the characteristics of the flankers (Experiments 2–4). Uncrowding in time occurs only if the flankers can potentially group away from the target for longer durations (Rectangles and Cubes; Experiment 1). Flankers that do not group away from the target for longer durations, such as Lines, do not provide any advantage for subsequent crowded stimuli. Hence, our preview effect reveals a very different type of mechanism than previous research.

Our results build on recent findings investigating temporal crowding (Harrison, Retell, Remington, & Mattingley, 2013; Hochmitz, Abu-Akel, & Yeshurun, 2024; Sahar & Yeshurun, 2023; Tkacz-Domb & Yeshurun, 2017, 2021; Yeshurun, Rashal, & Tkacz-Domb, 2015; see also Sayim et al., 2014), where the identification of a target object is impaired when distracting objects precede and follow it. Notably, while previous studies found that target recognition is impaired in the range of 500 ms (Hochmitz et al., 2024; Sahar & Yeshurun, 2023; Tkacz-Domb & Yeshurun, 2017; Tkacz-Domb & Yeshurun, 2021), our preview effect demonstrates that crowding can be reduced with flanker previews presented up to 1 s before the target. This apparent discrepancy could be explained by the difference in tasks and stimuli (oriented stimuli in Hochmitz et al., 2024; Sahar & Yeshurun, 2023; Tkacz-Domb & Yeshurun, 2021, or letters in Tkacz-Domb & Yeshurun, 2017; Yeshurun et al., 2015; Yeshurun & Rashal, 2010, vs. the vernier offset discrimination task in our study). Importantly, our paradigm involves spatial and temporal crowding components simultaneously, as flankers are positioned adjacent to the target in space as well as in time. Previous studies focused on purely temporal crowding without spatial crowding (Hochmitz et al., 2024; Sahar & Yeshurun, 2023; Tkacz-Domb & Yeshurun, 2017; Tkacz-Domb & Yeshurun, 2021; Yeshurun et al., 2015).

Finally, previous results show that crowding is not a fundamental bottleneck in vision (Herzog & Manassi, 2015; Manassi & Whitney, 2018). Our results take one step further in this respect, showing that target information is not irretrievably lost in the early stages of visual processing, as predicted by pooling mechanisms (Balas, Nakano, & Rosenholtz, 2009; Freeman & Simoncelli, 2011; Greenwood, Bex, & Dakin, 2009; Keshvari & Rosenholtz, 2016; Parkes, Lund, Angelucci, Solomon, & Morgan, 2001; Pelli, 2008; Rosenholtz, Yu, & Keshvari, 2019; Wilkinson et al., 1997). Target information loss under visual crowding conditions can be prevented, depending also on the temporal context (i.e., stimulus duration and time interval between preview and crowded stimulus).

## Conclusions

Our study sheds light on the intricate dynamics of crowding and uncrowding, revealing the important role of spatiotemporal grouping processes. Grouping of flankers requires a minimum stimulus duration to occur and can be initiated even with a 20-ms preview of the flankers. This preview effect, unlike standard priming effects, extends over large temporal distances, is dependent on the flankers’ configuration, and is gated by grouping mechanisms. Contrary to standard feedforward models of vision, our results are well explained by the LAMINART model, which implements recurrent, time-consuming mechanisms for segmenting visual scenes.

## References

[bib1] Bach, M. (1996). The Freiburg Visual Acuity Test—automatic measurement of visual acuity. *Optometry and Vision Science,* 73(1), 49–53. 8867682 10.1097/00006324-199601000-00008

[bib2] Balas, B., Nakano, L., & Rosenholtz, R. (2009). A summary-statistic representation in peripheral vision explains visual crowding. *Journal of Vision,* 9(12), 13, 10.1167/9.12.13. 20053104 PMC2923917

[bib3] Ballester, P., & Araujo, R. M. (2016). On the performance of GoogLeNet and AlexNet applied to sketches. *Proceedings of the AAAI Conference on Artificial Intelligence,* 30(1), 10.1609/aaai.v30i1.10171.

[bib4] Bornet, A., Choung, O. H., Doerig, A., Whitney, D., Herzog, M. H., & Manassi, M. (2021). Global and high-level effects in crowding cannot be predicted by either high-dimensional pooling or target cueing. *Journal of Vision,* 21(12), 1–25, 10.1167/jov.21.12.10. PMC8626847 34812839

[bib5] Bornet, A., Doerig, A., Herzog, M. H., Francis, G., & Van Der Burg, E. (2021). Shrinking Bouma's window: How to model crowding in dense displays. *PLoS Computational Biology,* 17(7), 1–14, 10.1371/journal.pcbi.1009187. PMC8284675 34228703

[bib6] Brainard, D. H. (1997). The Psychophysics Toolbox. *Spatial Vision,* 10, 433–436, http://www.psych.ucsb.edu/. 9176952

[bib7] Brendel, W., & Bethge, M. (2019). *Approximating CNNs with bag-of-local-features models works surprisingly well on ImageNet,* Published as a conference paper at the Seventh International Conference on Learning Representations (ICLR 2019), http://arxiv.org/abs/1904.00760.

[bib8] Chicherov, V., Plomp, G., & Herzog, M. H. (2014). Neural correlates of visual crowding. *NeuroImage,* 93(P1), 23–31, 10.1016/j.neuroimage.2014.02.021. 24582921

[bib9] Choung, O. H., Bornet, A., Doerig, A., & Herzog, M. H. (2021). Dissecting (un)crowding. *Journal of Vision,* 21(10), 10, 10.1167/jov.21.10.10. PMC8444456 34515740

[bib10] Choung, O. H., Rashal, E., Kunchulia, M., & Herzog, M. H. (2023). Specific Gestalt principles cannot explain (un)crowding. *Frontiers in Computer Science,* 5, 1–20, 10.1167/16.6.8.

[bib11] Chung, S. T. L. (2016). Spatio-temporal properties of letter crowding. *Journal of Vision,* 16(6), 1–20, 10.1167/16.6. PMC4898270 27088895

[bib12] Chung, S. T. L., & Patel, S. S. (2022). Spatial and temporal proximity of objects for maximal crowding. *Vision Research,* 194, 1–18, 10.1016/j.visres.2022.108012. 35042087

[bib13] Cichy, R. M., Khosla, A., Pantazis, D., Torralba, A., & Oliva, A. (2016). Comparison of deep neural networks to spatio-temporal cortical dynamics of human visual object recognition reveals hierarchical correspondence. *Scientific Reports,* 6, 1–13, 10.1038/srep27755. 27282108 PMC4901271

[bib14] Cichy, R. M., Pantazis, D., & Oliva, A. (2014). Resolving human object recognition in space and time. *Nature Neuroscience,* 17(3), 455–462, 10.1038/nn.3635. 24464044 PMC4261693

[bib15] Comaniciu, D., Meer, P., & Member, S. (2002). Mean shift: A robust approach toward feature space analysis. *IEEE Transactions on Pattern Analysis and Machine Intelligence,* 24(5), 603–619, 10.1109/34.1000236.

[bib16] DiCarlo, J. J., Zoccolan, D., & Rust, N. C. (2012). How does the brain solve visual object recognition? *Neuron,* 73(3), 415–434, 10.1016/j.neuron.2012.01.010. 22325196 PMC3306444

[bib17] Doerig, A., Schmittwilken, L., Sayim, B., Manassi, M., & Herzog, M. H. (2020). Capsule networks as recurrent models of grouping and segmentation. *PLoS Computational Biology,* 16(7), 1–19, 10.1371/journal.pcbi.1008017. PMC7394447 32692780

[bib18] Fabre-Thorpe, M., Delorme, A., Marlot, C., & Thorpe, S. (2001). A limit to the speed of processing in ultra-rapid visual categorization of novel natural scenes. *Journal of Cognitive Neuroscience,* 13(2), 171–180, http://mitprc.silverchair.com/jocn/article-pdf/13/2/171/1759015/089892901564234.pdf. 11244543 10.1162/089892901564234

[bib19] Felzenszwalb, P. F., & Huttenlocher, D. P. (2004). Efficient graph-based image segmentation _ enhanced reader. *International Journal of Computer Vision,* 59(2), 167–181.

[bib20] Francis, G., Grossberg, S., & Mingolla, E. (1994). Cortical dynamics of feature binding and reset: Control of visual persistence. *Vision Research,* 34(8), 1089–1104. 8160417 10.1016/0042-6989(94)90012-4

[bib21] Francis, G., Manassi, M., & Herzog, M. H. (2017). Neural dynamics of grouping and segmentation explain properties of visual crowding. *Psychological Review,* 124(4), 483–504, 10.1037/rev0000070. 28437128

[bib22] Freeman, J., & Simoncelli, E. P. (2011). Metamers of the ventral stream. *Nature Neuroscience,* 14(9), 1195–1204, 10.1038/nn.2889. 21841776 PMC3164938

[bib23] Geirhos, R., Rubisch, P., Michaelis, C., Bethge, M., Wichmann, F. A., & Brendel, W. (2018). *ImageNet-trained CNNs are biased towards texture; increasing shape bias improves accuracy and robustness,* Accepted at ICLR 2019 (oral), http://arxiv.org/abs/1811.12231.

[bib24] Gilbert, C. D., & Li, W. (2013). Top-down influences on visual processing. *Nature Reviews Neuroscience,* 14(5), 350–363, 10.1038/nrn3476. 23595013 PMC3864796

[bib25] Greenwood, J. A., Bex, P. J., & Dakin, S. C. (2009). Positional averaging explains crowding with letter-like stimuli. *Proceedings of the National Academy of Sciences,* 106(31), 13130–13135, www.pnas.org/cgi/content/full/. 10.1073/pnas.0901352106 PMC2711865 19617570

[bib26] Greenwood, J. A., Sayim, B., & Cavanagh, P. (2014). Crowding is reduced by onset transients in the target object (but not in the flankers). *Journal of Vision,* 14(6), 2, 10.1167/14.6.2. 25086085

[bib27] Harrison, W. J., & Bex, P. J. (2014). Integrating retinotopic features in spatiotopic coordinates. *Journal of Neuroscience,* 34(21), 7351–7360, 10.1523/JNEUROSCI.5252-13.2014. 24849367 PMC4028505

[bib28] Harrison, W. J., Retell, J. D., Remington, R. W., & Mattingley, J. B. (2013). Visual crowding at a distance during predictive remapping. *Current Biology,* 23(9), 793–798, 10.1016/j.cub.2013.03.050. 23562269

[bib29] He, K., Zhang, X., Ren, S., & Sun, J. (2016). Deep residual learning for image recognition. *IEEE Conference on Computer Vision and Pattern Recognition (CVPR),* Las Vegas, NV, USA, 770–778, 10.1109/CVPR.2016.90.

[bib30] Herzog, M. H. (2022). The irreducibility of vision: Gestalt, crowding and the fundamentals of vision. *Vision,* 6(2), 35, 10.3390/vision6020035. 35737422 PMC9228288

[bib31] Herzog, M. H., & Manassi, M. (2015). Uncorking the bottleneck of crowding: A fresh look at object recognition. *Current Opinion in Behavioral Sciences,* 1, 86–93, 10.1016/j.cobeha.2014.10.006.

[bib32] Herzog, M. H., Sayim, B., Chicherov, V., & Manassi, M. (2015). Crowding, grouping, and object recognition: A matter of appearance. *Journal of Vision,* 15(6), 1–18, 10.1167/15.6.5. PMC4429926 26024452

[bib33] Hilchey, M. D., Leber, A. B., & Pratt, J. (2018). Testing the role of response repetition in spatial priming in visual search. *Attention, Perception, and Psychophysics,* 80(6), 1362–1374, 10.3758/s13414-018-1550-7. 29949117

[bib34] Hochmitz, I., Abu-Akel, A., & Yeshurun, Y. (2024). Interference across time: Dissociating short from long temporal interference. *Frontiers in Psychology,* 15, 1–14, 10.3389/fpsyg.2024.1393065. PMC11305178 39114585

[bib35] Hubel, D. H., & Wiesel, A. T. N. (1962). Receptive fields, binocular interaction and functional architecture in the cat's visual cortex. *J. Phyiiol,* 160, 106. 10.1113/jphysiol.1962.sp006837 PMC1359523 14449617

[bib36] Huckauf, A., & Heller, D. (2004). On the relations between crowding and visual masking. *Perception and Psychophysics,* 66(4), 584–595. 15311658 10.3758/bf03194903

[bib37] Janiszewski, C., & Wyer, R. S. (2014). Content and process priming: A review. *Journal of Consumer Psychology,* 24(1), 96–118, 10.1016/j.jcps.2013.05.006.

[bib38] Jastrzębowska, M. A., Chicherov, V., Draganski, B., & Herzog, M. H. (2021). Unraveling brain interactions in vision: The example of crowding. *NeuroImage,* 240, 1–12, 10.1016/j.neuroimage.2021.118390. 34271157

[bib39] Keshvari, S., & Rosenholtz, R. (2016). Pooling of continuous features provides a unifying account of crowding. *Journal of Vision,* 16(3), 1–15, 10.1167/16.3.39. PMC4790193 26928055

[bib40] Kim, T., & Pasupathy, A. (2024). Neural correlates of crowding in macaque area V4. *Journal of Neuroscience,* 44(24), 1–16, 10.1523/JNEUROSCI.2260-23.2024. PMC11170949 38670806

[bib41] Kirchner, H., & Thorpe, S. J. (2006). Ultra-rapid object detection with saccadic eye movements: Visual processing speed revisited. *Vision Research,* 46(11), 1762–1776, 10.1016/j.visres.2005.10.002. 16289663

[bib42] Kon, M., & Francis, G. (2022). Perceptual grouping strategies in a letter identification task: Strategic connections, selection, and segmentation. *Attention, Perception, and Psychophysics,* 84(6), 1944–1963, 10.3758/s13414-022-02515-1. 35701661

[bib43] Kristjánsson, Á., & Campana, G. (2010). Where perception meets memory: A review of repetition priming in visual search tasks. *Attention, Perception, and Psychophysics,* 72(1), 5–18, 10.3758/APP.72.1.5. 20045875

[bib44] Kristjánsson, Á., & Driver, J. (2008). Priming in visual search: Separating the effects of target repetition, distractor repetition and role-reversal. *Vision Research,* 48(10), 1217–1232, 10.1016/j.visres.2008.02.007. 18374961

[bib45] Kristjánsson, Á., Wang, D., & Nakayama, K. (2002). The role of priming in conjunctive visual search. *Cognition,* 85(1), 37–52, 10.1016/S0010-0277(02)00074-4. 12086712

[bib46] Krizhevsky, A., Sutskever, I., & Hinton, G. E. (2012). ImageNet Classification with deep convolutional neural networks. *Advances in Neural Information Processing Systems,* 25, 1–9, http://code.google.com/p/cuda-convnet/.

[bib47] Lecun, Y., Bottou, E., Bengio, Y., & Haffner, P. (1998). Gradient-based learning applied to document recognition. *Proceedings of the IEEE,* 86(11), 2278–2324, 10.1109/5.726791.

[bib48] Levi, D. M. (2008). Crowding—an essential bottleneck for object recognition: A mini-review. *Vision Research,* 48(5), 635–654, 10.1016/j.visres.2007.12.009. 18226828 PMC2268888

[bib49] Levi, D. M., Klein, S. A., & Aitsebaomo, A. P. (1985). Vernier acuity, crowding and cortical magnification. *Vision Research,* 25(7), 963–977. 4049746 10.1016/0042-6989(85)90207-x

[bib50] Malania, M., Herzog, M. H., & Westheimer, G. (2007). Grouping of contextual elements that affect vernier thresholds. *Journal of Vision,* 7(2), 1–7, 10.1167/7.2.1. 18217816

[bib51] Manassi, M., France, B. S., & Herzog, M. H. (2012). Grouping, pooling, and when bigger is better in visual crowding. *Journal of Vision,* 12(10), 1–14, 10.1167/12.10.13. 23019118

[bib52] Manassi, M., Hermens, F., Francis, G., & Herzog, M. H. (2015). Release of crowding by pattern completion. *Journal of Vision,* 15(8), 1–15, 10.1167/15.8.16. 26114679

[bib53] Manassi, M., Lonchampt, S., Clarke, A., & Herzog, M. H. (2016). What crowding can tell us about object representations. *Journal of Vision,* 16(3), 1–13, 10.1167/16.3.35. 26913627

[bib54] Manassi, M., Sayim, B., & Herzog, M. H. (2013). When crowding of crowding leads to uncrowding. *Journal of Vision,* 13(13), 1–10, 10.1167/13.13.10. 24213598

[bib55] Manassi, M., & Whitney, D. (2018). Multi-level crowding and the paradox of object recognition in clutter. *Current Biology,* 28(3), R127–R133, 10.1016/j.cub.2017.12.051. 29408262

[bib56] Parkes, L., Lund, J., Angelucci, A., Solomon, J. A., & Morgan, M. (2001). Compulsory averaging of crowded orientation signals in human vision. *Nature Neuroscience,* 4(7), 739–744, 10.1038/89532. 11426231

[bib57] Pelli, D. G. (2008). Crowding: a cortical constraint on object recognition. *Current Opinion in Neurobiology,* 18(4), 445–451, 10.1016/j.conb.2008.09.008. 18835355 PMC3624758

[bib58] Phillips, W. A. (1974). On the distinction between sensory storage and short-term visual memory. *Perception & Psychophysics,* 16(2), 283–290.

[bib59] Ren, X., & Malik, J. (2003). Learning a classification model for segmentation. In *Proceedings of the Ninth IEEE International Conference on Computer Vision (ICCV'03)*. 0-7695-1950-4/03 2003 IEEE.

[bib60] Ricci, M., & Serre, T. (2020). Hierarchical models of the visual system. In Jaeger D., & Jung R. (eds.). *Encyclopedia of computational neuroscience* (pp. 1–14). New York, NY: Springer, 10.1007/978-1-4614-7320-6_345-2.

[bib61] Riesenhuber, M., & Poggio, T. (1999). Hierarchical models of object recognition in cortex. *Nature Neuroscience,* 2(11), 1–25, http://neurosci.nature.com. 10526343 10.1038/14819

[bib62] Rosenholtz, R., Yu, D., & Keshvari, S. (2019). Challenges to pooling models of crowding: Implications for visual mechanisms. *Journal of Vision,* 19(7), 15, 10.1167/jov.19.7.15. PMC6660188 31348486

[bib63] Rousselet, G. A., Macé, M. J. M., & Fabre-Thorpe, M. (2004). Animal and human faces in natural scenes: How specific to human faces is the N170 ERP component? *Journal of Vision,* 4(1), 13–21, 10.1167/4.1.2. 14995895

[bib64] Saarela, T. P., Sayim, B., Westheimer, G., & Herzog, M. H. (2009). Global stimulus configuration modulates crowding. *Journal of Vision,* 9(2), 1–11, 10.1167/9.2.5. 19271915

[bib65] Saarela, T. P., Westheimer, G., & Herzog, M. H. (2010). The effect of spacing regularity on visual crowding. *Journal of Vision,* 10(10), 1–7, 10.1167/10.10.17. 20884482

[bib66] Sahar, T., & Yeshurun, Y. (2023). Temporal crowding with central vision reveals the fragility of visual representations. *Journal of Experimental Psychology: General,* 153(2), 339–351, 10.1037/xge0001496. 37956076

[bib67] Sayim, B., Manassi, M., & Herzog, M. (2014). How color, regularity, and good Gestalt determine backward masking. *Journal of Vision,* 14(7), 1–11, 10.1167/14.7.8. 24944237

[bib68] Sayim, B., Westheimer, G., & Herzog, M. H. (2010). Gestalt factors modulate basic spatial vision. *Psychological Science,* 21, 641–644, 10.1177/0956797610368811i. 20483840

[bib69] Schwetlick, L., Manassi, M., Herzog, M. H., & Francis, G. (2025). Does surface completion fail to support uncrowding? *Journal of Vision,* 25(3), 1, 10.1167/JOV.25.3.1. PMC11887933 40029235

[bib70] Scolari, M., Kohnen, A., Barton, B., & Awh, E. (2007). Spatial attention, preview, and popout: Which factors influence critical spacing in crowded displays? *Journal of Vision,* 7(2), 1–23, 10.1167/7.2.7. 18217822

[bib71] Shi, J., & Malik, J. (2000). Normalized Cuts and Image Segmentation. *IEEE Transactions on Pattern Analysis and Machine Intelligence,* 22(8), 888–905.

[bib72] Simonyan, K., & Zisserman, A. (2014). *Very deep convolutional networks for large-scale image recognition,* 1–14, http://arxiv.org/abs/1409.1556.

[bib73] Soo, L., Chakravarthi, R., & Andersen, S. K. (2018). Critical resolution: A superior measure of crowding. *Vision Research,* 153, 13–23, 10.1016/j.visres.2018.08.005. 30240717 PMC6294650

[bib74] Sperling, G. (1960). The information available in brief visual presentations. *Psychological Monographs: General and Applied,* 74(11), 1–29.

[bib75] Strasburger, H., Rentschler, I., & Jüttner, M. (2011). Peripheral vision and pattern recognition: A review. *Journal of Vision,* 11(5), 1–84, 10.1167/11.5.13. PMC11073400 22207654

[bib76] Taylor, M. M., & Creelman, C. D. (1967). PEST: Efficient estimates on probability functions. *The Journal of the Acoustical Society of America,* 41(4A), 782–787, 10.1121/1.1910407.

[bib77] Theeuwes, J. (2013). Feature-based attention: It is all bottom-up priming. *Philosophical Transactions of the Royal Society B: Biological Sciences,* 368(1628), 1–11, 10.1098/rstb.2013.0055. PMC3758198 24018717

[bib78] Thorpe, S., Fize, D., & Marlot, C. (1996). Speed of processing in the human visual system. *Nature,* 381, 520–522. 8632824 10.1038/381520a0

[bib79] Tiurina, N. A., Markov, Y. A., Choung, O. H., Herzog, M. H., & Pascucci, D. (2022). Unlocking crowding by ensemble statistics. *Current Biology,* 32(22), 4975–4981.e3, 10.1016/j.cub.2022.10.003. 36309011

[bib80] Tkacz-Domb, S., & Yeshurun, Y. (2017). Spatial attention alleviates temporal crowding, but neither temporal nor spatial uncertainty are necessary for the emergence of temporal crowding. *Journal of Vision,* 17(3), 1–12, 10.1167/17.3.9. 28282481

[bib81] Tkacz-Domb, S., & Yeshurun, Y. (2021). Temporal crowding is a unique phenomenon reflecting impaired target encoding over large temporal intervals. *Psychonomic Bulletin & Review,* 28, 1885–1893, 10.3758/s13423-021-01943-8/Published. 34080137

[bib82] VanRullen, R., & Thorpe, S. J. (2001). The time course of visual processing: From early perception to decision-making. *Journal of Cognitive Neuroscience,* 13(4), 454–461, http://direct.mit.edu/jocn/article-pdf/13/4/454/1933828/08989290152001880.pdf. 11388919 10.1162/08989290152001880

[bib83] Wallace, J. M., Chiu, M. K., Nandy, A. S., & Tjan, B. S. (2013). Crowding during restricted and free viewing. *Vision Research,* 84, 50–59, 10.1016/j.visres.2013.03.010. 23563172 PMC3665516

[bib84] Wallis, T. S., Funke, C. M., Ecker, A. S., Gatys, L. A., Wichmnn, F. A., & Bethge, M. (2019). Image content is more important than Bouma's Law for scene metamers. *ELife,* 8, 1–43 10.7554/eLife.42512.001. PMC6491040 31038458

[bib85] Westheimer, G., & Hauske, G. (1975). Temporal and spatial interference with vernier acuity. *Vision Research,* 15, 1137–1141. 1166614 10.1016/0042-6989(75)90012-7

[bib86] Whitney, D., & Levi, D. M. (2011). Visual crowding: A fundamental limit on conscious perception and object recognition. *Trends in Cognitive Sciences,* 15(4), 160–168, 10.1016/j.tics.2011.02.005. 21420894 PMC3070834

[bib87] Wilkinson, F., Wilson, H. R., & Ellemberg, D. (1997). Lateral interactions in peripherally viewed texture arrays. *Journal of the Optical Society of America. A, Optics, Image Science, and Vision* 14(9), 2057–2068, 10.1364/josaa.14.002057. 9291601

[bib88] Yeshurun, Y., & Rashal, E. (2010). Precueing attention to the target location diminishes crowding and reduces the critical distance. *Journal of Vision,* 10(10), 1–12, 10.1167/10.10.16. 20884481

[bib89] Yeshurun, Y., Rashal, E., & Tkacz-Domb, S. (2015). Temporal crowding and its interplay with spatial crowding. *Journal of Vision,* 15(3), 1–16, 10.1167/15.3.11. 25788705

